# Selective Prescribing of First‐Line SGLT2 Inhibitor and GLP‐1 RA in Adults With Severe Mental Illness and Type 2 Diabetes

**DOI:** 10.1002/pds.70510

**Published:** 2026-10-18

**Authors:** Yueh‐Yi Chiang, Chixiang Chen, Xiaojuan Li, Charmaine D. Rochester‐Eyeguokan, Wendy Camelo Castillo, Susan dosReis

**Affiliations:** ^1^ Department of Practice, Sciences, and Health Outcomes Research University of Maryland School of Pharmacy Baltimore Maryland USA; ^2^ Division of Biostatistics and Bioinformatics, Department of Medicine University of Maryland School of Medicine Baltimore Maryland USA; ^3^ Department of Population Medicine Harvard Medical School and Harvard Pilgrim Health Care Institute Boston Massachusetts USA

**Keywords:** channeling bias, first‐line, GLP‐1 receptor agonist, selective prescribing, severe mental illness, SGLT2 inhibitor

## Abstract

**Purpose:**

SGLT2 inhibitors (SGLT2i) and GLP‐1 receptor agonists (GLP‐1 RA) provide cardiovascular (CV) benefits, but their first‐line use among adults with severe mental illness (SMI) is not well characterized. We evaluated selective prescribing of SGLT2i, GLP‐1 RA, and metformin among adults with SMI and type 2 diabetes (T2D).

**Methods:**

Using 2017–2022 Medicare claims, we identified adults with SMI and T2D who initiated first‐line SGLT2i, GLP‐1 RA, or metformin during 1/1/2018–12/31/2021. By calendar year, standardized mean differences (SMD) assessed baseline characteristic differences, and propensity score overlap coefficients (OVL) assessed group comparability between SGLT2i or GLP‐1 RA initiators and metformin initiators.

**Results:**

We identified 1322 SGLT2i, 2306 GLP‐1 RA, and 21 144 metformin initiators. From 2018 to 2021, SGLT2i initiators shifted from younger to older than metformin initiators (SMD, −0.11 to 0.19), while differences in CVD (0.10 to 0.50) and CKD (0.18 to 0.62) increased. GLP‐1 RA initiators consistently had higher prevalence of obesity (SMD, 0.33 to 0.41) and CKD (0.42 to 0.28) than metformin initiators. Compared with metformin initiators, both SGLT2i and GLP‐1 RA initiators had lower prevalence of schizophrenia, antipsychotic use, and recent psychiatrist visits but higher prevalence of major depressive disorder; these differences generally persisted over time. OVL increased from 42.7% to 50.7% for SGLT2i and from 42.3% to 50.0% for GLP‐1 RA versus metformin.

**Conclusions:**

First‐line SGLT2i and GLP‐1 RA prescribing among adults with SMI was selective according to cardiometabolic and mental health‐related characteristics. Future comparative studies should account for these differences and temporal prescribing changes to mitigate confounding.

## Key Points

- First‐line SGLT2i and GLP‐1 RA prescribing among adults with SMI and T2D differed systematically from metformin prescribing.
- SGLT2i initiators increasingly represented older adults and individuals with CVD and CKD from 2018 through 2021.
- GLP‐1 RA initiators consistently had a higher prevalence of obesity and CKD than metformin initiators.
- SGLT2i and GLP‐1 RA initiators had lower prevalence of schizophrenia, antipsychotic use, and recent psychiatrist visits than metformin initiators.
- Limited propensity score overlap and temporal changes in treatment selection should be considered in future comparative studies.

## Plain Language Summary

Newer diabetes medicines called SGLT2 inhibitors and GLP‐1 receptor agonists can help reduce cardiovascular risk. This may be especially important for adults with severe mental illness, who have a high burden of heart disease and other cardiometabolic conditions. However, little is known about how these medicines are selected as first diabetes treatment in this population. We studied adults with severe mental illness and type 2 diabetes who started an SGLT2 inhibitor, GLP‐1 receptor agonist, or metformin between 2018 and 2021. Over time, people starting SGLT2 inhibitors became older and were increasingly more likely to have cardiovascular disease and chronic kidney disease than those starting metformin. People starting GLP‐1 receptor agonists consistently had more obesity and chronic kidney disease. Mental health characteristics also differed: people starting either newer medicine were less likely to have schizophrenia, use antipsychotic medicines, or have recently seen a psychiatrist than those starting metformin. These findings show that both cardiometabolic and mental health characteristics may influence which diabetes medicine is selected first. Future studies comparing these treatments should account for these differences to avoid biased conclusions.

## 1 Introduction

Severe mental illness (SMI), defined as schizophrenia, bipolar disorder, and major depressive disorder [1], is associated with substantial burden of physical illness [2]. Individuals with SMI have a 15‐ to 20‐year reduction in life expectancy [3], with much of the excess mortality attributable to premature cardiovascular disease (CVD) [4]. Adults with SMI have a 2‐ to 4‐fold higher prevalence of CVD risk factors (e.g., diabetes, hypertension, dyslipidemia, and metabolic syndrome) than the general population [5]. High rates of health‐risk behaviors, social disadvantage, and metabolic adverse effects of psychotropic medications may further increase cardiometabolic risk [6, 7].

Over the past decade, clinical trials have demonstrated cardiovascular benefits of two novel classes of antihyperglycemic agents in adults with or without type 2 diabetes (T2D). Sodium‐glucose cotransporter‐2 inhibitors (SGLT2i) reduce the risk of major adverse cardiovascular events and hospitalization for heart failure, while glucagon‐like peptide‐1 receptor agonists (GLP‐1 RA) reduce the risk of stroke, myocardial infarction, and death [8, 9, 10, 11]. These findings have led to guideline updates and expanded indications for their use beyond glycemic control [12, 13].

Despite these advances, evidence on how newer antihyperglycemic agents have been incorporated into diabetes care for adults with SMI remains limited. Individuals with SMI are less likely than those without SMI to receive recommended diabetes monitoring and may experience poorer quality of care [14, 15]. Studies have also found lower use of recommended cardiovascular medications in individuals with SMI versus without SMI, suggesting potential gaps in cardiovascular risk management [16]. Metformin has long been the preferred first‐line treatment for T2D [17], and clinical guidance supports managing diabetes in individuals with SMI according to established diabetes treatment standards [18]. A Danish registry based study found that individuals with SMI and T2D were more likely to receive antihyperglycemic treatment, particularly metformin, than those without SMI [XX]. However, little is known about the use of SGLT2i and GLP‐1 RA as first‐line treatment among adults with SMI, particularly how prescribing patterns have evolved with their expanded use in the general T2D population.

Understanding first‐line treatment selection is important because adults with SMI may particularly benefit from the cardiometabolic effects of SGLT2i and GLP‐1 RA but may also face barriers to their use. Clinical factors, socioeconomic circumstances, prescriber‐related factors, and health insurance policies, may influence the uptake of these newer agents and contribute to selective prescribing (i.e., channeling) [19, 20, 21]. To date, evidence is lacking on whether SGLT2i and GLP‐1 RA are selectively prescribed as first‐line treatment according to patient characteristics within the SMI population.

To address this gap, we investigated first‐line antihyperglycemic prescribing patterns among adults with SMI and comorbid T2D, focusing on selective prescribing. We characterized adults who initiated SGLT2i, GLP‐1 RA, or metformin from 2018 through 2021 and compared baseline CVD and other chronic conditions across initiation groups. We hypothesized that, over time, SGLT2i and GLP‐1 RA initiators would have a higher prevalence of cardiovascular risk factors than metformin initiators.

## 2 Methods

### 2.1 Data Sources

We used Medicare fee‐for‐service administrative claims from the Centers for Medicare & Medicaid Services (CMS) Chronic Conditions Data Warehouse (CCW) for January 1, 2017, through December 31, 2022, representing a 20% random sample of Medicare beneficiaries. Medicare is the US federal health insurance program for adults aged 65 years or older and for younger individuals with qualifying disabilities or conditions. A unique beneficiary identifier enabled linkage across data files for person‐level health care utilization histories. Medical claims include dates, care settings, and associated diagnosis and procedure codes. Part D prescription drug event claims capture dispensing transactions, including dispensing date, National Drug Code, generic names, and days' supply. Demographic and enrollment characteristics were obtained from the Master Beneficiary Summary File.

### 2.2 Study Design and Study Cohort

We implemented a new‐user design to identify a retrospective cohort of individuals with SMI and comorbid T2D. Eligible individuals initiated first‐line monotherapy with an SGLT2i, a GLP‐1 RA, or metformin from January 1, 2018 through December 31, 2021. The index date (Day 0) was the first dispensing for the study drug. First‐line monotherapy was the initiation of a single study drug (SGLT2i, GLP‐1 RA, or metformin) with no dispensing of any antihyperglycemic agents—including metformin, SGLT2i, GLP‐1 RA, dipeptidyl peptidase‐4 inhibitors, sulfonylureas, or thiazolidinediones—during the 365‐day baseline period (Day −365 to Day −1). Generic names of the study drugs are listed in Table S1.

Individuals were included if they (1) had continuous Medicare enrollment with complete medical and pharmacy benefits (Parts A, B, and D) during the 365‐day baseline period before the index date; (2) were aged 18 years or older on the index date; and (3) had evidence of SMI and T2D during baseline and/or on the index date. SMI was defined by at least one International Classification of Diseases, 10th Revision, Clinical Modification (ICD‐10‐CM) diagnosis code for schizophrenia, bipolar disorder, or major depressive disorder. Individuals could meet the criteria for more than one SMI condition (e.g., an individual could have both schizophrenia and major depressive disorder). In the absence of a single validated claims‐based algorithm for SMI, we used CMS CCW definitions [22]. Operational definitions for SMI and T2D are provided in Table S2.

We excluded individuals who (1) initiated more than one antihyperglycemic drug class on the index date; (2) had evidence of type 1 diabetes, gestational diabetes, polycystic ovary syndrome, organ transplant, end‐stage renal disease (ESRD), or HIV/AIDS during baseline and/or on the index date; or (3) had ESRD as the current or original reason for Medicare entitlement. These conditions were excluded because they may represent contraindications to the study drugs or complex clinical circumstances that could introduce confounding.

### 2.3 Baseline Characteristics

We measured baseline characteristics from Day −365 through Day 0 (the index date), including sociodemographic characteristics, comorbidities, medication use, specialist visits in the 7 days before the index date, and health care resource utilization (HCRU). SMI‐related clinical characteristics included diagnoses of schizophrenia, bipolar disorder, and major depressive disorder; mental health comorbidities; psychotropic medications; and psychiatry visits in the 7 days before the index date. We used quartiles of the Social Vulnerability Index (SVI) as an area‐level measure of social vulnerability, with higher quartiles indicating greater vulnerability [23]. Variables were selected a priori informed by published literature on factors associated with antihyperglycemic treatment initiation and cardiovascular outcomes [24, 25]. Operational definitions for all baseline measures are provided in Table S3.

### 2.4 Statistical Analysis

Descriptive statistics summarized cohort characteristics overall and by index drug. We assessed between‐group differences using chi‐squared tests for categorical variables and ANOVA for continuous variables. We considered two‐sided *p* < 0.05 statistically significant. To evaluate temporal changes in characteristics of individuals who initiated an SGLT2i, GLP–1 RA, or metformin, we stratified the study period into four calendar‐year time blocks: T1 (January 1–December 31, 2018), T2 (January 1–December 31, 2019), T3 (January 1–December 31, 2020), and T4 (January 1–December 31, 2021). These time blocks aligned with guideline updates, cardiovascular outcomes trial publications, and US Food and Drug Administration (FDA) drug label changes that may have influenced prescribing of SGLT2i and GLP‐1 RA. Table S4 summarizes key events from 2018 through 2021 that may have affected prescribing.

To assess selective prescribing, we conducted pairwise comparisons using metformin as the reference group (SGLT2i vs. metformin; GLP‐1 RA vs. metformin). For each calendar year, we calculated standardized mean differences (SMD) in baseline CVD risk factors. Positive SMDs indicated that the prevalence (or mean values) of a given characteristic was higher among SGLT2i or GLP‐1 RA initiators than among metformin initiators, whereas negative SMDs indicated that the prevalence (or mean values) was higher among metformin initiators than among SGLT2i or GLP‐1 RA initiators. By convention, an absolute SMD < 0.1 was considered to indicate a negligible difference between groups. We plotted SMDs across the four time blocks for selected CVD risk factors and indicators of disease severity. To assess temporal trends, we regressed SMDs on time block (coded 0–3) using ordinary least squares. We considered 2‐sided *p* < 0.05 statistically significant.

Within each time block, we estimated propensity scores (PS) to summarize differences in baseline characteristics across treatment groups and to evaluate overlap (i.e., potential clinical equipoise). We used logistic regression to estimate the probability of treatment initiation, with metformin as the reference group, and included baseline characteristics specified in Table S3. We assessed overlap of PS distributions using the overlapping coefficient (OVL), which quantifies the degree of overlap between groups' PS distributions [26]. An OVL close to 100% indicates substantial common support and greater comparability between groups. We conducted all analyses in SAS Studio version 9.4 (SAS Institute). This study was deemed exempt by the Institutional Review Board at the University of Maryland, Baltimore (HP‐00112780).

## 3 Results

### 3.1 Study Cohort Characteristics

We identified adults with SMI and T2D who initiated first‐line SGLT2i (*n* = 1322), GLP‐1 RA (*n* = 2306), or metformin (*n* = 21 144) from January 1, 2018, through December 31, 2021 (Table S5). Baseline characteristics are shown in Table 1. Most were aged ≥ 50 (*n* = 27 972; 88.7%), female (*n* = 15 256; 61.6%), and White (*n* = 19 389; 78.3%). More than half originally qualified for Medicare through disability insurance benefits (*n* = 13 844; 55.9%). The cohort had a high prevalence of obesity (*n* = 13 137; 53.0%), CVD (*n* = 14 924; 60.2%), hyperlipidemia (*n* = 20 451; 82.6%), and hypertension (*n* = 22 177; 89.5%). The SMI cohort mainly represented individuals with major depressive disorder (*n* = 20 487; 82.7%). Over half had an anxiety disorder (*n* = 14 364; 58.0%). Psychotropic use in this cohort included antidepressants (*n* = 19 580; 79.0%), antipsychotics (*n* = 7704; 31.1%), and to a lesser extent mood stabilizers (*n* = 3823; 15.4%). In the 7 days before the index date, primary care visits were more common than psychiatrist visits (71.2% vs. 5.8%). Most individuals (*n* = 20 335; 82.1%) resided in areas within SVI quartiles 2 through 4, representing higher levels of social vulnerability. Baseline characteristics differed across treatment groups for most prespecified variables (*p* < 0.05; Table 1).

**TABLE 1:** Baseline characteristics of first‐line antihyperglycemic medication initiators, overall and by index drug.

*N* ^a^	Study cohort	SGLT2i	GLP‐1 RA	Metformin	*p*
	24 772	1322	2306	21 144	
Demographics					
Age, mean (SD)	66.2 (12.9)	67.1 (12.1)	65.0 (12.1)	66.3 (13.0)	< 0.0001
Age group, *n* (%)					
18–49	2800 (11.3)	111 (8.4)	265 (11.5)	2424 (11.5)	0.0028
50–69	11 270 (45.5)	619 (46.8)	1174 (50.9)	9477 (44.8)	< 0.0001
≥ 70	10 702 (43.2)	592 (44.8)	867 (37.6)	9243 (43.7)	< 0.0001
Sex, *n* (%)					
Male	9516 (38.4)	537 (40.6)	712 (30.9)	8267 (39.1)	< 0.0001
Female	15 256 (61.6)	785 (59.4)	1594 (69.1)	12 877 (60.9)	< 0.0001
Race, *n* (%)					
White	19 389 (78.3)	1039 (78.6)	1843 (79.9)	16 507 (78.1)	0.1176
Black	3273 (13.2)	153 (11.6)	303 (13.1)	2817 (13.3)	0.1889
Other^b^	1781 (7.2)	107 (8.1)	129 (5.6)	1545 (7.3)	0.004
Unknown	329 (1.3)	23 (1.7)	31 (1.3)	275 (1.3)	0.3992
Geographic region, *n* (%)					
Northeast	4699 (19.0)	NR^c^	NR^c^	NR^c^	0.2896
Midwest	6148 (24.8)	NR^c^	NR^c^	NR^c^	0.0082
South	9666 (39.0)	NR^c^	NR^c^	NR^c^	0.0369
West	4235 (17.1)	NR^c^	NR^c^	NR^c^	< 0.0001
Other	24 (0.1)	NR^c^	NR^c^	NR^c^	0.2525
Index year, *n* (%)					
2018	7719 (31.2)	220 (16.6)	624 (27.1)	6875 (32.5)	< 0.0001
2019	6887 (27.8)	295 (22.3)	627 (27.2)	5965 (28.2)	< 0.0001
2020	5211 (21.0)	306 (23.1)	490 (21.2)	4415 (20.9)	0.1411
2021	4955 (20.0)	501 (37.9)	565 (24.5)	3889 (18.4)	< 0.0001
SVI, *n* (%)					
Quartile 1: 0–0.2500	4437 (17.9)	225 (17.0)	424 (18.4)	3788 (17.9)	0.5858
Quartile 2: 0.2501–0.5000	6354 (25.6)	351 (26.6)	588 (25.5)	5415 (25.6)	0.738
Quartile 3: 0.5001–0.7500	8122 (32.8)	436 (33.0)	775 (33.6)	6911 (32.7)	0.6614
Quartile 4: 0.7501–1.0	5859 (23.7)	310 (23.4)	519 (22.5)	5030 (23.8)	0.3817
Dual eligibility status, *n* (%)					
No duals	11 609 (46.9)	673 (50.9)	983 (42.6)	9953 (47.1)	< 0.0001
Partial duals	2518 (10.2)	156 (11.8)	296 (12.8)	2066 (9.8)	< 0.0001
Full duals	10 645 (43.0)	493 (37.3)	1027 (44.5)	9125 (43.2)	< 0.0001
LIS status, *n* (%)					
No LIS	10 217 (41.2)	590 (44.6)	836 (36.3)	8791 (41.6)	< 0.0001
Partial LIS	926 (3.7)	65 (4.9)	100 (4.3)	761 (3.6)	0.014
Full LIS	13 629 (55.0)	667 (50.5)	1370 (59.4)	11 592 (54.8)	< 0.0001
Current entitlement reason, *n* (%)					
Old age and survivor's insurance	15 708 (63.4)	865 (65.4)	1382 (59.9)	13 461 (63.7)	0.0006
Disability insurance benefits	9064 (36.6)	457 (34.6)	924 (40.1)	7683 (36.3)	0.0006
Original entitlement reason, *n* (%)					
Old age and survivor's insurance	10 928 (44.1)	602 (45.5)	889 (38.6)	9437 (44.6)	< 0.0001
Disability insurance benefits	13 844 (55.9)	720 (54.5)	1417 (61.4)	11 707 (55.4)	< 0.0001
SMI, *n* (%)					
Schizophrenia	4692 (18.9)	142 (10.7)	267 (11.6)	4283 (20.3)	< 0.0001
Bipolar disorder	5082 (20.5)	211 (16.0)	426 (18.5)	4445 (21.0)	< 0.0001
Depressive disorder	20 487 (82.7)	1169 (88.4)	2023 (87.7)	17 295 (81.8)	< 0.0001
Comorbidities, *n* (%)					
Obesity	13 137 (53.0)	769 (58.2)	1573 (68.2)	10 795 (51.1)	< 0.0001
Diabetic nephropathy	8221 (33.2)	675 (51.1)	1090 (47.3)	6456 (30.5)	< 0.0001
Diabetic neuropathy	7758 (31.3)	616 (46.6)	1208 (52.4)	5934 (28.1)	< 0.0001
Diabetic retinopathy	2289 (9.2)	219 (16.6)	445 (19.3)	1625 (7.7)	< 0.0001
CVD	14 924 (60.2)	977 (73.9)	1487 (64.5)	12 460 (58.9)	< 0.0001
AMI	1376 (5.6)	163 (12.3)	134 (5.8)	1079 (5.1)	< 0.0001
AF and flutter	4281 (17.3)	344 (26.0)	429 (18.6)	3508 (16.6)	< 0.0001
HF and non‐IHD	6653 (26.9)	586 (44.3)	761 (33.0)	5306 (25.1)	< 0.0001
IHD	8571 (34.6)	693 (52.4)	890 (38.6)	6988 (33.0)	< 0.0001
Stroke/transient ischemic attack	3857 (15.6)	220 (16.6)	324 (14.1)	3313 (15.7)	0.0685
PVD	6830 (27.6)	457 (34.6)	721 (31.3)	5652 (26.7)	< 0.0001
Hyperlipidemia	20 451 (82.6)	1190 (90.0)	1968 (85.3)	17 293 (81.8)	< 0.0001
Hypertension	22 177 (89.5)	1236 (93.5)	2143 (92.9)	18 798 (88.9)	< 0.0001
Hypothyroidism	7952 (32.1)	459 (34.7)	810 (35.1)	6683 (31.6)	0.0003
CKD	6801 (27.5)	601 (45.5)	1000 (43.4)	5200 (24.6)	< 0.0001
COPD	8735 (35.3)	520 (39.3)	835 (36.2)	7380 (34.9)	0.0029
Cancer (any type)	2913 (11.8)	173 (13.1)	293 (12.7)	2447 (11.6)	0.0845
Anxiety disorders	14 364 (58.0)	778 (58.9)	1341 (58.2)	12 245 (57.9)	0.7872
PTSD	1745 (7.0)	80 (6.1)	200 (8.7)	1465 (6.9)	0.0028
AUD	1693 (6.8)	65 (4.9)	84 (3.6)	1544 (7.3)	< 0.0001
DUD	3803 (15.4)	194 (14.7)	328 (14.2)	3281 (15.5)	0.2049
OUD	1949 (7.9)	110 (8.3)	213 (9.2)	1626 (7.7)	0.0266
TUD	6461 (26.1)	332 (25.1)	509 (22.1)	5620 (26.6)	< 0.0001
Medications, *n* (%)					
Antithrombotic medications	6569 (26.5)	532 (40.2)	735 (31.9)	5302 (25.1)	< 0.0001
ACE inhibitors or ARBs	13 943 (56.3)	881 (66.6)	1445 (62.7)	11 617 (54.9)	< 0.0001
Beta blockers	11 631 (47.0)	789 (59.7)	1235 (53.6)	9607 (45.4)	< 0.0001
Calcium‐channel blockers	7834 (31.6)	406 (30.7)	761 (33.0)	6667 (31.5)	0.2705
Diuretics	11 666 (47.1)	786 (59.5)	1289 (55.9)	9591 (45.4)	< 0.0001
Statins	16 203 (65.4)	972 (73.5)	1604 (69.6)	13 627 (64.4)	< 0.0001
Insulin	6389 (25.8)	684 (51.7)	1545 (67.0)	4160 (19.7)	< 0.0001
Antipsychotics	7704 (31.1)	295 (22.3)	580 (25.2)	6829 (32.3)	< 0.0001
Mood stabilizers	3823 (15.4)	153 (11.6)	317 (13.7)	3353 (15.9)	< 0.0001
Antidepressants	19 580 (79.0)	1058 (80.0)	1901 (82.4)	16 621 (78.6)	< 0.0001
Physician specialties, *n* (%)					
Cardiologist	2641 (10.7)	266 (20.1)	126 (5.5)	2249 (10.6)	< 0.0001
Endocrinologist	1215 (4.9)	123 (9.3)	307 (13.3)	785 (3.7)	< 0.0001
Primary care	17 632 (71.2)	847 (64.1)	1473 (63.9)	15 312 (72.4)	< 0.0001
Psychiatrist	1436 (5.8)	33 (2.5)	60 (2.6)	1343 (6.4)	< 0.0001
HCRU					
Any hospitalizations, *n* (%)	11 810 (47.7)	636 (48.1)	943 (40.9)	10 231 (48.4)	< 0.0001
Any ED visits, *n* (%)	16 079 (64.9)	853 (64.5)	1443 (62.6)	13 783 (65.2)	0.0426
Any outpatient encounters, *n* (%)	24 772 (100.0)	1322 (100.0)	2306 (100.0)	21 144 (100.0)	—
No. of hospitalizations, mean (SD)	1.0 (1.7)	1.1 (1.7)	0.8 (1.4)	1.0 (1.7)	< 0.0001
No. of ED visits, mean (SD)	2.2 (4.0)	2.1 (3.4)	2.0 (3.3)	2.2 (4.1)	0.0035
No. of outpatient encounters, mean (SD)	58.4 (45.4)	65.6 (47.9)	63.4 (44.7)	57.4 (45.2)	< 0.0001
No. of unique medications, mean (SD)	16.1 (7.8)	18.3 (7.9)	19.5 (8.4)	15.6 (7.6)	< 0.0001

Abbreviations: ACE inhibitors or ARBs, angiotensin‐converting enzyme inhibitors or angiotensin receptor blockers; AF, atrial fibrillation; AMI, acute myocardial infarction; AUD, alcohol use disorder; CKD, chronic kidney disease; COPD, chronic obstructive pulmonary disease; CVD, cardiovascular disease; DUD, drug use disorder; ED, emergency department; HCRU, health care resource utilization; HF, heart failure; IHD, ischemic heart disease; LIS, low‐income subsidy; OUD, opioid use disorder; PTSD, post‐traumatic stress disorder; PVD, peripheral vascular disease; SD, standard deviation; SMI, severe mental illness; SVI, Social Vulnerability Index; TUD, tobacco use disorder.

^a^ Due to rounding, percentages may not total 100.0%.

^b^ The “Other” race category includes Asian, Hispanic, North American Native, and other races.

^c^ NR indicates not reportable because the cell count was fewer than 11 in the “Other” category.

### 3.2 Pairwise Comparison of Baseline Characteristics by Calendar‐Year

Baseline characteristics by calendar‐year are in Tables S6–S7, and SMD trends are in Figure 1 and Figure S1. We observed temporal changes comparing annual cohorts of initiators from 2018 through 2021.

**FIGURE 1:**
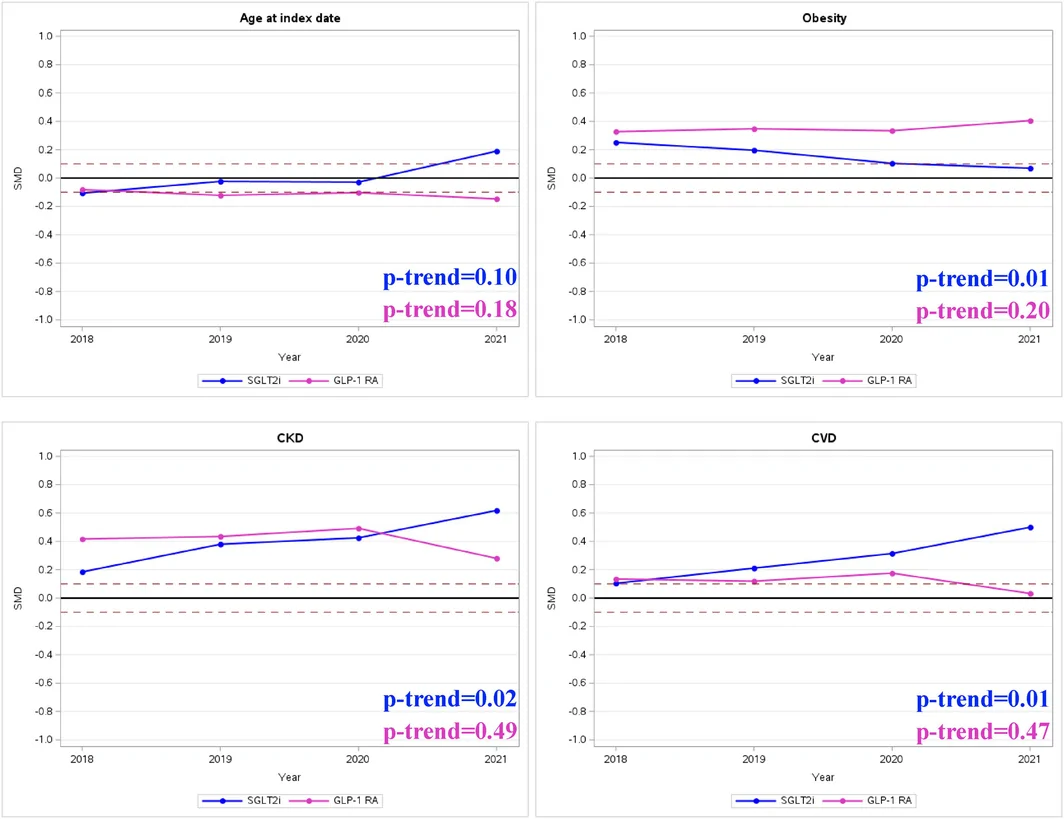
Trends in standardized mean differences for selected baseline characteristics, 2018–2021. CKD, chronic kidney disease; CVD, cardiovascular disease; HF, heart failure; SMD, standardized mean differences. (A) SMDs were calculated pairwise, comparing SGLT2i versus metformin and GLP‐1 RA versus metformin, with metformin as the reference group. (B) Positive SMDs indicate higher prevalence (or mean values) among SGLT2i (or GLP‐1 RA) initiators; negative SMDs indicate higher prevalence (or mean values) among metformin initiators. (C) An absolute SMD < 0.1 was considered to indicate a negligible difference between groups. (D) We defined comorbidities based on diagnoses recorded during the 365‐day baseline period; we defined specialty visits as any visit to the specified specialist within 7 days before the index date.

#### 3.2.1 SGLT2i Versus Metformin

In 2018, SGLT2i initiators were younger (SMD = −0.11) and had a higher prevalence of obesity (SMD = 0.25), diabetic nephropathy (SMD = 0.20), CVD (SMD = 0.10), and chronic kidney disease (CKD; SMD = 0.18) than metformin initiators. SGLT2i initiators had a similar prevalence of cardiologist visits in the 7 days before the index date (SMD = 0.03) and a lower prevalence of baseline hospitalizations (SMD = −0.16). SGLT2i initiators had a lower prevalence of schizophrenia (SMD = −0.21), antipsychotic use (SMD = −0.14), mood stabilizer use (SMD = −0.11), and psychiatrist visits in the 7 days before the index date (SMD = −0.15), but a higher prevalence of major depressive disorder (SMD = 0.17).

Age differences reversed over time: in 2018, SGLT2i initiators were younger than metformin initiators (mean, 64.2 vs. 65.5 years; SMD = −0.11), whereas in 2021 they were older (mean, 69.4 vs. 67.0 years; SMD = 0.19). SMDs increased over time for diabetic nephropathy (SMD, 0.20 in 2018 vs. 0.60 in 2021; *p* = 0.02), CVD (0.10 vs. 0.50; *p* = 0.01), and CKD (0.18 vs. 0.62; *p* = 0.02), but decreased for obesity (0.25 vs. 0.07; *p* = 0.01). By 2021, SGLT2i initiators also had a higher prevalence of cardiologist visits (SMD, 0.03 in 2018 vs. 0.49 in 2021; *p* = 0.13) and hospitalizations (−0.16 vs. 0.14; *p* = 0.06) than metformin initiators, although these temporal trends were not statistically significant. Differences in SMI‐related characteristics generally persisted through 2021, including a lower prevalence of schizophrenia (SMD = −0.33), antipsychotic use (SMD = −0.32), mood stabilizer use (SMD = −0.17), and recent psychiatrist visits (SMD = −0.17), and a higher prevalence of major depressive disorder (SMD = 0.22). However, none of these SMI‐related differences changed significantly over time.

#### 3.2.2 GLP‐1 RA Versus Metformin

In 2018, GLP‐1 RA initiators had a similar mean age to metformin initiators (SMD = −0.08) but a higher prevalence of obesity (SMD = 0.33), diabetic nephropathy (SMD = 0.35), CVD (SMD = 0.13), CKD (SMD = 0.42), and insulin use (SMD = 1.17). GLP‐1 RA initiators also had a higher prevalence of endocrinologist visits in the 7 days before the index date (SMD = 0.40) and a lower prevalence of baseline hospitalizations (SMD = −0.12). GLP‐1 RA initiators had a lower prevalence of schizophrenia (SMD = −0.26), alcohol use disorder (SMD = −0.17), antipsychotic use (SMD = −0.17), mood stabilizer use (SMD = −0.12), and recent psychiatrist visits (SMD = −0.19), but a higher prevalence of major depressive disorder (SMD = 0.24) and antidepressant use (SMD = 0.15).

From 2018 to 2021, GLP‐1 RA initiators remained younger than metformin initiators, with a widening age difference (2018: mean, 64.5 vs. 65.5 years; SMD = −0.08; 2021: mean, 65.2 vs. 67.0 years; SMD = −0.15). SMDs were similar between 2018 and 2021 for obesity (0.33 vs. 0.41), diabetic nephropathy (0.35 vs. 0.22), CKD (0.42 vs. 0.28), and insulin use (1.17 vs. 0.87), whereas the imbalance in CVD decreased (0.13 vs. 0.03). In 2021, GLP‐1 RA initiators continued to have a higher prevalence of endocrinologist visits (SMD, 0.40 in 2018 vs. 0.30 in 2021) and a lower prevalence of hospitalizations (−0.12 vs. −0.23) than metformin initiators. Differences in several SMI‐related characteristics persisted through 2021, including a lower prevalence of schizophrenia (SMD = −0.26), alcohol use disorder (SMD = −0.16), antipsychotic use (SMD = −0.13), and recent psychiatrist visits (SMD = −0.16), and a higher prevalence of major depressive disorder (SMD = 0.13). None of these SMI‐related differences changed significantly over time. Only endocrinologist and primary care physician visits showed statistically significant trends over time (*p* < 0.05).

### 3.3 PS Overlap by Calendar‐Year

Figure 2 shows PS distributions for the SGLT2i versus metformin comparison by calendar year. In 2018, metformin initiators' PS values were concentrated between 0.0 and 0.05, indicating a low estimated probability of SGLT2i initiation. By 2021, the PS distribution among metformin initiators shifted upward to approximately 0.1–0.2, indicating higher estimated probabilities of SGLT2i initiation. We observed a similar shift for the GLP‐1 RA versus metformin comparison (Figure 3). For SGLT2i versus metformin, the OVLs were 42.7% (2018), 48.0% (2019), 51.0% (2020), and 50.7% (2021). For GLP‐1 RA versus metformin, the corresponding OVLs were 42.3% (2018), 43.4% (2019), 41.5% (2020), and 50.0% (2021). Summary statistics for PS values are provided in Table S8.

**FIGURE 2:**
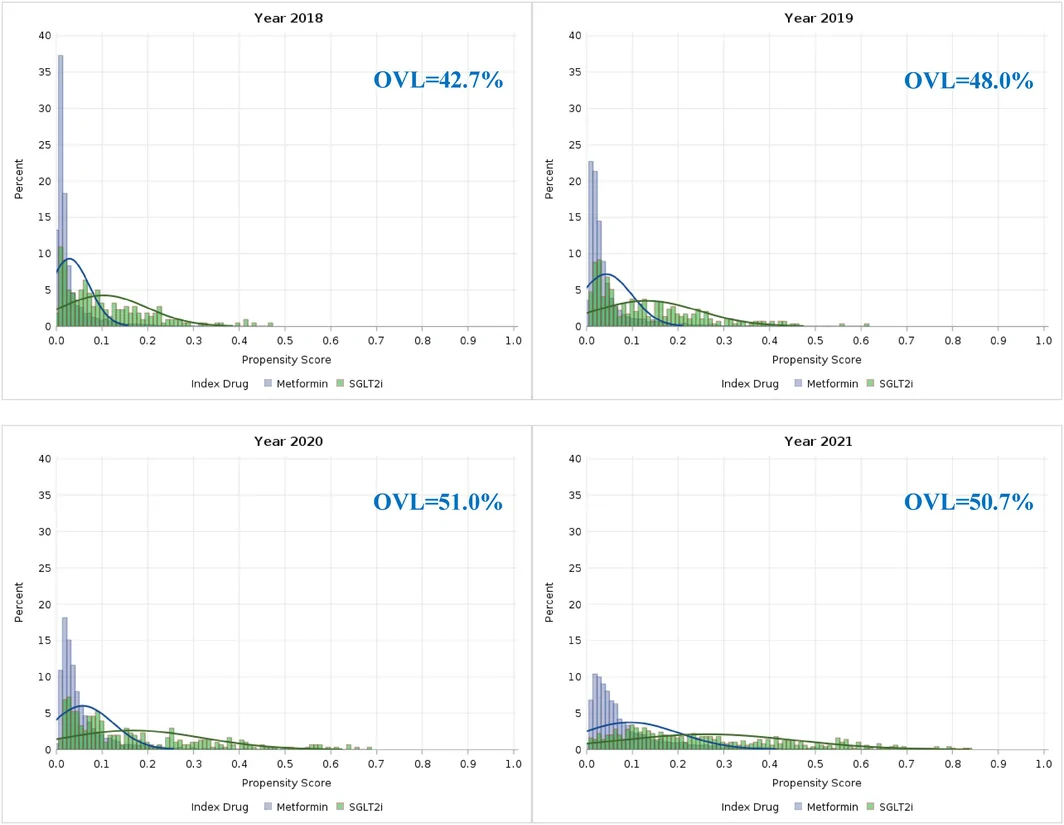
Propensity score distributions over time for SGLT2i versus metformin initiators, 2018–2021. OVL, overlap coefficient.

**FIGURE 3:**
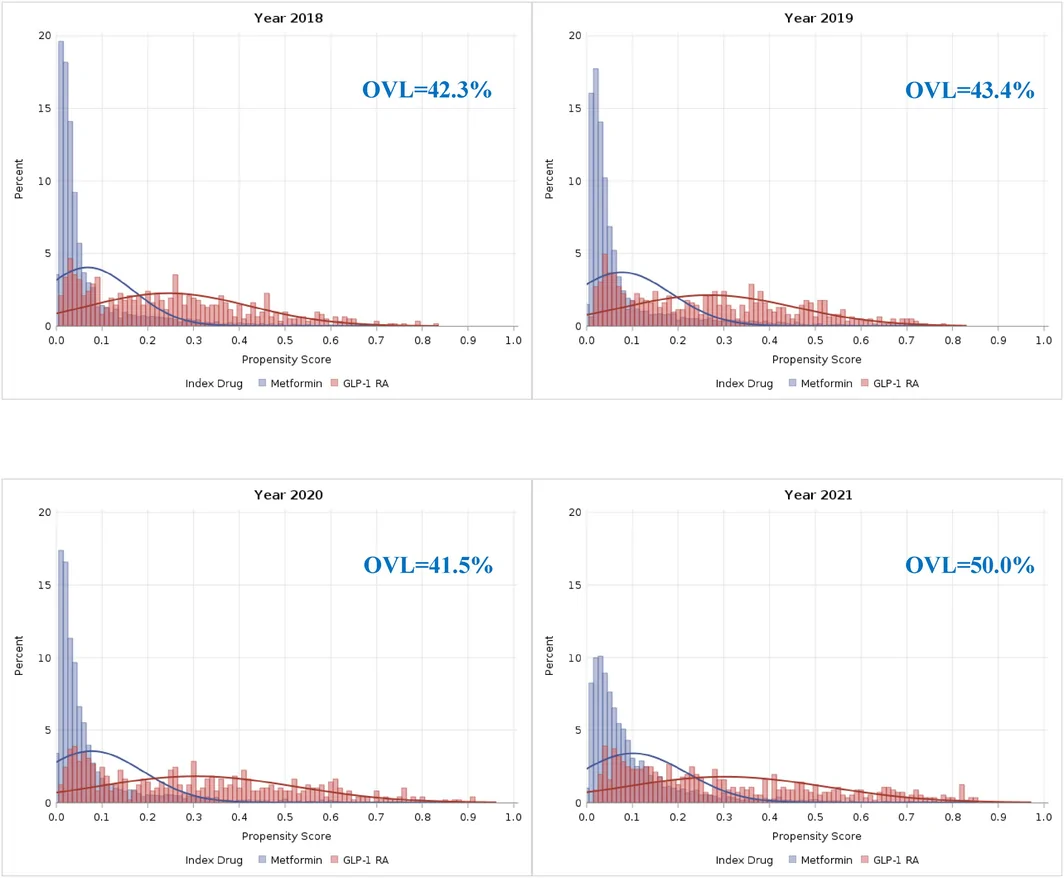
Propensity score distributions over time for GLP‐1 RA versus metformin initiators, 2018–2021. OVL, overlap coefficient.

## 4 Discussion

This study found evolving selective prescribing of SGLT2i and GLP‐1 RA among adults with SMI and comorbid T2D, reflected by systematic between‐group differences in baseline characteristics that shifted over time. Overall, SGLT2i and GLP‐1 RA initiators had a higher comorbidity burden, more microvascular complications, and greater baseline insulin use than metformin initiators, suggesting more advanced and/or uncontrolled diabetes. Over time, SGLT2i were increasingly prescribed to older adults and those with diabetic nephropathy, CKD, HF, and CVD, whereas GLP‐1 RA were consistently prescribed to adults with CKD and obesity. Differences in mental health related characteristics were also evident. Compared with metformin initiators, SGLT2i and GLP‐1 RA initiators had a lower prevalence of schizophrenia, antipsychotic use, and recent psychiatrist visits but a higher prevalence of major depressive disorder. These differences generally persisted over time. Although PS overlap increased modestly, it remained limited, indicating persistent selective prescribing.

Our findings share several patterns with first‐line SGLT2i prescribing in the broader T2D population [27]. Shin et al. found that SGLT2i initiators were initially younger than metformin initiators, but this age difference narrowed over time. They also found that CVD and recent cardiologist visits became more prevalent among SGLT2i initiators over time. We observed similar patterns. In our study, SGLT2i initiators shifted from being younger than metformin initiators in 2018 to older in 2021. CKD, CVD, HF, and recent cardiologist visits also became more prevalent among SGLT2i initiators over time. These similarities suggest that emerging clinical evidence, regulatory approvals, and guideline recommendations influenced first‐line SGLT2i prescribing among adults with SMI in ways broadly consistent with prescribing in the general T2D population.

Our findings also identify treatment‐selection patterns that may be particularly relevant to the SMI population. We found that individuals with schizophrenia, antipsychotic treatment, substance use disorders, or greater involvement in mental health care were less likely to initiate SGLT2i and GLP‐1 RA as first‐line treatment. Notably, antipsychotic use was less prevalent among GLP‐1 RA initiators despite the potential weight‐related benefits of GLP‐1 RA for individuals experiencing antipsychotic‐induced weight gain [28]. Mental illness severity, challenges in managing complex medication regimens, and fragmentation between mental and somatic health care may contribute to these patterns [19, 29, 30]. However, we could not measure these factors directly; therefore, we cannot determine if they explain the observed prescribing patterns. Studies incorporating detailed clinical measures and patient and prescriber perspectives are needed to better understand how mental health characteristics influence diabetes treatment selection.

To investigate overall between‐group differences, we examined PS distributions separately for each calendar year from 2018 through 2021. Although PS overlap increased modestly, it remained around 50% in 2021, indicating limited comparability between treatment groups. Limited PS overlap suggests that future comparative studies should carefully consider the choice and application of PS methods, such as matching or weighting, to improve comparability between treatment groups. In addition, the observed temporal shifts in patient characteristics suggest that calendar time should be explicitly addressed. Sequential cohort designs with time‐stratified PS estimation may help account for changes in prescribing over time [31, 32]. PS methods can then be applied within each time stratum to improve covariate balance.

This study has several strengths. To our knowledge, it is the first to characterize first‐line initiation of SGLT2i, GLP‐1 RA, and metformin specifically among adults with SMI and comorbid T2D. We evaluated a comprehensive set of clinical and mental health characteristics and assessed temporal shifts in selective prescribing. PS distributions provided summary measures of between‐group comparability and may inform control confounding in future observational studies. Finally, nationwide Medicare claims allowed us to examine a large, clinically complex population of Medicare‐eligible adults with SMI. This study has limitations. We assessed prescribing at the drug‐class level and did not examine agent‐specific patterns. Therefore, some temporal changes may reflect evidence emerging for specific agents within a class. In addition, our cohort identification period ended in 2021, preventing assessment of more recent prescribing patterns. Because uptake of newer medications may occur gradually after new evidence, indications, or guideline changes, studies using more recent data are needed to determine whether these prescribing patterns have continued to evolve.

## 5 Conclusion

Among adults with SMI and comorbid T2D, characteristics of SGLT2i initiators shifted over time toward individuals with higher baseline CVD risk. In contrast, characteristics of GLP‐1 RA initiators consistently differed from those of metformin initiators, with differences remaining relatively stable from 2018 to 2021. Our findings suggest that mental health‐related clinical profiles and care contexts may also influence first‐line antihyperglycemic selection. Future observational studies in adults with SMI and comorbid T2D should account for both cardiometabolic and mental health‐related factors that contribute to selective prescribing to minimize confounding and support valid causal inference.

## Author Contributions

Yueh‐Yi Chiang and Susan dosReis conceptualized the study and its design; all authors contributed to the methodology. Yueh‐Yi Chiang and Susan dosReis acquired the data. Yueh‐Yi Chiang analyzed the data. All authors contributed to the interpretation of the results. Susan dosReis supervised the study. Yueh‐Yi Chiang drafted the initial manuscript. Susan dosReis, Chixiang Chen, Xiaojuan Li, Charmaine D. Rochester‐Eyeguokan, and Wendy Camelo Castillo critically reviewed and revised the manuscript for important intellectual content. Yueh‐Yi Chiang and Susan dosReis are the guarantors of this work and, as such, had full access to all the data in the study and take responsibility for the integrity of the data and the accuracy of the data analysis.

## Funding

The authors have nothing to report.

## Conflicts of Interest

Xiaojuan Li has received support from the National Institute on Aging (grant K01AG073651) during the conduct of the study. Susan dosReis has received grants from the National Institute of Mental Health, PCORI, PhRMA Foundation, PhRMA, and GSK; and consultant with Alexion; all unrelated to the submitted study. No other potential conflicts of interest relevant to this article were reported.

## Supporting information


**Table S1:** Medications (generic names) included in the study.
**Table S2:** Algorithms for defining severe mental illness and type 2 diabetes.
**Table S3:** Operational definitions for baseline measures.
**Table S4:** Overview of evidence and regulatory updates on the cardiovascular benefits of newer antihyperglycemic agents, 2018–2021.
**Table S5:** Cohort selection attrition table.
**Table S6:** Baseline characteristics of first‐line antihyperglycemic medication initiators, stratified by calendar year: SGLT2i versus metformin.
**Table S7:** Baseline characteristics of first‐line antihyperglycemic medication initiators, stratified by calendar year: GLP‐1 RA versus metformin.
**Table S8:** Propensity score summary statistics, 2018–2021.
**Figure S1:** Trends in standardized mean differences for selected baseline characteristics, 2018–2021.

## Data Availability

The data that support the findings of this study are available from CMS Chronic Conditions Data Warehouse. Restrictions apply to the availability of these data, which were used under license for this study. Data are available from the author(s) with the permission of CMS Chronic Conditions Data Warehouse.

## References

[1] National Institute of Mental Health (NIMH) , “Mental Illness,” accessed December 19, 2025, https://www.nimh.nih.gov/health/statistics/mental‐illness.

[2] D. Pizzol , M. Trott , L. Butler , et al., “Relationship Between Severe Mental Illness and Physical Multimorbidity: A Meta‐Analysis and Call for Action,” BMJ Mental Health 26, no. 1 (2023): e300870, 10.1136/bmjment-2023-300870. PMC10619039 37907331

[3] J. K. N. Chan , C. U. Correll , C. S. M. Wong , et al., “Life Expectancy and Years of Potential Life Lost in People With Mental Disorders: A Systematic Review and Meta‐Analysis,” EClinicalMedicine 65 (2023): 102294, 10.1016/j.eclinm.2023.102294. PMC10641487 37965432

[4] M. Goldfarb , M. De Hert , J. Detraux , et al., “Severe Mental Illness and Cardiovascular Disease,” Journal of the American College of Cardiology 80, no. 9 (2022): 918–933, 10.1016/j.jacc.2022.06.017. 36007991

[5] C. Polcwiartek , K. O'Gallagher , D. J. Friedman , et al., “Severe Mental Illness: Cardiovascular Risk Assessment and Management,” European Heart Journal 45, no. 12 (2024): 987–997, 10.1093/eurheartj/ehae054. PMC10972692 38538149

[6] V. Vaccarino , E. Prescott , A. J. Shah , et al., “Mental Health Disorders and Their Impact on Cardiovascular Health Disparities,” Lancet Regional Health—Europe 56 (2025): 101373, 10.1016/j.lanepe.2025.101373. PMC12587323 41200014

[7] V. Androshchuk , N. Montarello , and R. Rajani , “Adverse Cardiovascular Effects of Psychotropic Medications,” British Journal of Hospital Medicine 86, no. 4 (2025): 1–18, 10.12968/hmed.2024.0773. 40265538

[8] B. Zinman , C. Wanner , J. M. Lachin , et al., “Empagliflozin, Cardiovascular Outcomes, and Mortality in Type 2 Diabetes,” New England Journal of Medicine 373, no. 22 (2015): 2117–2128, 10.1056/NEJMoa1504720. 26378978

[9] S. D. Wiviott , I. Raz , M. P. Bonaca , et al., “Dapagliflozin and Cardiovascular Outcomes in Type 2 Diabetes,” New England Journal of Medicine 380, no. 4 (2019): 347–357, 10.1056/NEJMoa1812389. 30415602

[10] S. P. Marso , G. H. Daniels , K. Brown‐Frandsen , et al., “Liraglutide and Cardiovascular Outcomes in Type 2 Diabetes,” New England Journal of Medicine 375, no. 4 (2016): 311–322, 10.1056/NEJMoa1603827. PMC4985288 27295427

[11] H. C. Gerstein , H. M. Colhoun , G. R. Dagenais , et al., “Dulaglutide and Cardiovascular Outcomes in Type 2 Diabetes (REWIND): A Double‐Blind, Randomised Placebo‐Controlled Trial,” Lancet 394, no. 10193 (2019): 121–130, 10.1016/S0140-6736(19)31149-3. 31189511

[12] American Diabetes Association , “Standards of Medical Care in Diabetes—2018 Abridged for Primary Care Providers,” Clinical Diabetes 36, no. 1 (2018): 14–37, 10.2337/cd17-0119. PMC5775000 29382975

[13] U.S. FDA , Approves INVOKANA (Canagliflozin) to Reduce the Risk of Heart Attack, Stroke or Cardiovascular Death in Adults With Type 2 Diabetes and Established Cardiovascular Disease (JNJ.com, 2018), accessed April 4, 2025, https://www.jnj.com/media‐center/press‐releases/u‐s‐fda‐approves‐invokana‐canagliflozin‐to‐reduce‐the‐risk‐of‐heart‐attack‐stroke‐or‐cardiovascular‐death‐in‐adults‐with‐type‐2‐diabetes‐and‐established‐cardiovascular‐disease.

[14] E. Wagner , M. Højlund , J. G. Fiedorowicz , et al., “Disparities in Diabetes Treatment and Monitoring for People With and Without Mental Disorders: A Systematic Review and Meta‐Analysis,” Lancet Psychiatry 13, no. 2 (2026): 112–124, 10.1016/S2215-0366(25)00332-3. 41506273

[15] C. Bakkedal , F. Persson , M. Kriegbaum , et al., “Diabetes Treatment for Persons With Severe Mental Illness: A Registry‐Based Cohort Study to Explore Medication Treatment Differences for Persons With Type 2 Diabetes With and Without Severe Mental Illness,” PLoS One 18, no. 6 (2023): e0287017, 10.1371/journal.pone.0287017. PMC10263345 37310947

[16] M. Solmi , J. Fiedorowicz , L. Poddighe , et al., “Disparities in Screening and Treatment of Cardiovascular Diseases in Patients With Mental Disorders Across the World: Systematic Review and Meta‐Analysis of 47 Observational Studies,” American Journal of Psychiatry 178 (2021): 793–803, 10.1176/appi.ajp.2021.21010031. 34256605

[17] C. Baker , C. Retzik‐Stahr , V. Singh , R. Plomondon , V. Anderson , and N. Rasouli , “Should Metformin Remain the First‐Line Therapy for Treatment of Type 2 Diabetes?,” Therapeutic Advances in Endocrinology and Metabolism 12 (2021): 2042018820980225, 10.1177/2042018820980225. PMC7809522 33489086

[18] S. J. Cooper , G. P. Reynolds , With expert co‐authors (in alphabetical order) , et al., “BAP Guidelines on the Management of Weight Gain, Metabolic Disturbances and Cardiovascular Risk Associated With Psychosis and Antipsychotic Drug Treatment,” Journal of Psychopharmacology 30, no. 8 (2016): 717–748, 10.1177/0269881116645254. 27147592

[19] C. E. Blixen , S. Kanuch , A. T. Perzynski , C. Thomas , N. V. Dawson , and M. Sajatovic , “Barriers to Self‐Management of Serious Mental Illness and Diabetes,” American Journal of Health Behavior 40, no. 2 (2016): 194–204, 10.5993/AJHB.40.2.4. PMC4928189 26931751

[20] A. Yaseen and S. W. Lahiri , “Health Care Provider Prescribing Habits and Barriers to Use of New Type 2 Diabetes Medications: A Single‐System Survey Study,” Clinical Diabetes: A Publication of the American Diabetes Association 41, no. 4 (2023): 490–501, 10.2337/cd22-0100. PMC10577502 37849520

[21] J. Luo , R. Feldman , S. D. Rothenberger , I. Hernandez , and W. F. Gellad , “Coverage, Formulary Restrictions, and Out‐Of‐Pocket Costs for Sodium‐Glucose Cotransporter 2 Inhibitors and Glucagon‐Like Peptide 1 Receptor Agonists in the Medicare Part D Program,” JAMA Network Open 3, no. 10 (2020): e2020969, 10.1001/jamanetworkopen.2020.20969. PMC7563069 33057641

[22] Home , “Chronic Conditions Data Warehouse,” accessed April 4, 2025, https://www2.ccwdata.org.

[23] CDC , “Social Vulnerability Index. Place and Health—Geospatial Research, Analysis, and Services Program (GRASP),” October 22, 2024, accessed August 21, 2026, https://www.atsdr.cdc.gov/place‐health/php/svi/index.html.

[24] H. Shin , S. Schneeweiss , R. J. Glynn , and E. Patorno , “Cardiovascular Outcomes in Patients Initiating First‐Line Treatment of Type 2 Diabetes With Sodium‐Glucose Cotransporter‐2 Inhibitors Versus Metformin: A Cohort Study,” Annals of Internal Medicine 175, no. 7 (2022): 927–937, 10.7326/M21-4012. 35605236

[25] Y. Xie , B. Bowe , H. Xian , T. Loux , J. B. McGill , and Z. Al‐Aly , “Comparative Effectiveness of SGLT2 Inhibitors, GLP‐1 Receptor Agonists, DPP‐4 Inhibitors, and Sulfonylureas on Risk of Major Adverse Cardiovascular Events: Emulation of a Randomised Target Trial Using Electronic Health Records,” Lancet Diabetes and Endocrinology 11, no. 9 (2023): 644–656, 10.1016/S2213-8587(23)00171-7. 37499675

[26] S. V. Belitser , E. P. Martens , W. R. Pestman , R. H. Groenwold , A. de Boer , and O. H. Klungel . “Measuring Balance and Model Selection in Propensity Score Methods,” Pharmacoepidemiol Drug Safety 20, no. 11 (2011): 1115–1129, 10.1002/pds.2188. 21805529

[27] H. Shin , S. Schneeweiss , R. J. Glynn , and E. Patorno , “Evolving Channeling in Prescribing SGLT‐2 Inhibitors as First‐Line Treatment for Type 2 Diabetes,” Pharmacoepidemiology and Drug Safety 31, no. 5 (2022): 566–576, 10.1002/pds.5406. PMC8989653 34985178

[28] S. Jacobson , N. Margolese , and H. C. Margolese , “GLP‐1 Receptor Agonists as a Novel Solution for Antipsychotic‐Induced Weight Gain in Severe and Persistent Mental Illness,” Canadian Journal of Psychiatry. Revue Canadienne de Psychiatrie 71, no. 6 (2026): 413–419, 10.1177/07067437251386626. PMC12528064 41091899

[29] J. F. Farley , R. A. Hansen , M. E. Domino , et al., “Continuity of Medication Management in Medicaid Patients With Chronic Comorbid Conditions: An Examination by Mental Health Status,” General Hospital Psychiatry 45 (2017): 25–31, 10.1016/j.genhosppsych.2016.12.001. PMC5843714 28274335

[30] T. Dorey , M. Haddad , H. McBain , K. Mulligan , and A. Zarska , “A Systematic Review of Barriers and Enablers That Health Professionals Experience to the Delivery of Type 2 Diabetes Care for Adults With Severe Mental Illness,” Diabetic Medicine 40, no. 7 (2023): e15098, 10.1111/dme.15098. 36995359

[31] S. Schneeweiss , J. J. Gagne , R. J. Glynn , M. Ruhl , and J. A. Rassen , “Assessing the Comparative Effectiveness of Newly Marketed Medications: Methodological Challenges and Implications for Drug Development,” Clinical Pharmacology and Therapeutics 90, no. 6 (2011): 777–790, 10.1038/clpt.2011.235. 22048230

[32] S. Schneeweiss , C. Gopalakrishnan , D. B. Bartels , et al., “Sequential Monitoring of the Comparative Effectiveness and Safety of Dabigatran in Routine Care,” Circulation. Cardiovascular Quality and Outcomes 12, no. 2 (2019): e005173, 10.1161/CIRCOUTCOMES.118.005173. 30764655

