# Supplementary material for: Selective Prescribing of First‐Line SGLT2 Inhibitor and GLP‐1 RA in Adults With Severe Mental Illness and Type 2 Diabetes
Source: Pharmacoepidemiol Drug Saf. 2026 Oct 18;35(11):e70510. doi: 10.1002/pds.70510 (PMC13677212; doi:10.1002/pds.70510)
Supplement: Supplementary file 1 [file PDS-35-e70510-s001.docx]

**Online-Only Supplemental Material**

Selective Prescribing of First-Line SGLT2 Inhibitor and GLP-1 RA in Adults with Severe Mental Illness and Type 2 Diabetes

Yueh-Yi Chiang, BS; Chixiang Chen, PhD; Xiaojuan Li, PhD; Charmaine D. Rochester-Eyeguokan, PharmD; Wendy Camelo Castillo, PhD; Susan dosReis, PhD

| Supplemental Table S1. | Medications (Generic Names) Included in the Study |
| --- | --- |
| Supplemental Table S2. | Algorithms for Defining Severe Mental Illness and Type 2 Diabetes |
| Supplemental Table S3. | Operational Definitions for Baseline Measures |
| Supplemental Table S4. | Overview of Evidence and Regulatory Updates on the Cardiovascular Benefits of Newer Antihyperglycemic Agents, 2018–2021 |
| Supplemental Table S5. | Cohort Selection Attrition Table |
| Supplemental Table S6. | Baseline Characteristics of First-Line Antihyperglycemic Medication Initiators, Stratified by Calendar Year: SGLT2i vs Metformin |
| Supplemental Table S7. | Baseline Characteristics of First-Line Antihyperglycemic Medication Initiators, Stratified by Calendar Year: GLP-1 RA vs Metformin |
| Supplemental Table S8. | Propensity Score Summary Statistics, 2018–2021 |
| Supplemental Figure S1. | Trends in Standardized Mean Differences for Selected Baseline Characteristics, 2018–2021 |

**Supplemental Table S1. Medications (Generic Names) Included in the Study**

| **Drug Class** | **Generic Names** |
| --- | --- |
| SGLT2i | canagliflozin, dapagliflozin, empagliflozin, ertugliflozin |
| GLP-1 RA | albiglutide, dulaglutide, exenatide, liraglutide, lixisenatide, semaglutide  Note: We did not include tirzepatide because of its dual mechanistic function as both a glucose-dependent insulinotropic polypeptide receptor agonist and a GLP-1 RA. |
| Metformin | metformin |

**Supplemental Table S2. Algorithms for Defining Severe Mental Illness and Type 2 Diabetes**

| **Diagnosis** | **ICD-10 Codes (Any DX on the Claim)** | **Number/Type of Claims to Qualify** |
| --- | --- | --- |
| Schizophrenia and Other Psychotic Disorders | F06.0, F06.2, F20.0, F20.1, F20.2, F20.3, F20.5, F20.81, F20.89, F20.9, F21, F22, F23, F24, F25.0, F25.1, F25.8, F25.9, F28, F29, F32.3, F33.3, F44.89 | At least 1 inpatient claim OR 2 other non-drug claims of any service type^a,b^ |
| Bipolar Disorder | F30.10, F30.11, F30.12, F30.13, F30.2, F30.3, F30.4, F30.8, F30.9, F31.0, F31.10, F31.11, F31.12, F31.13, F31.2, F31.30, F31.31, F31.32, F31.4, F31.5, F31.60, F31.61, F31.62, F31.63, F31.64, F31.70, F31.71, F31.72, F31.73, F31.74, F31.75, F31.76, F31.77, F31.78, F31.81, F31.89, F31.9, F33.8, F34.81, F34.89, F34.9, F39 | At least 1 inpatient claim OR 2 other non-drug claims of any service type^a,b^ |
| Major Depressive Disorders | F32.0, F32.1, F32.2, F32.3, F32.4, F32.5, F32.89, F32.9, F32.A, F33.0, F33.1, F33.2, F33.3, F33.40, F33.41, F33.42, F33.8, F33.9, F34.1 | At least 1 inpatient claim OR 2 other non-drug claims of any service type^a,b^ |
| Type 2 Diabetes | E08.X, E09.X, E11.X, and E13.X (Exclude: E13.65) | At least 1 inpatient/SNF/HHA  claim OR 2 HOP/carrier  claims^b^ |

Abbreviations: DX, diagnosis; HHA, home health agency; HOP, hospital outpatient; SNF, skilled nursing facility.

a Non-drug claims do not include Medicare Part D Event or Medicaid MAX RX claims.
b When two claims are required, they must occur at least one day apart (i.e., different claim through dates).

References: Chronic Conditions - Chronic Conditions Data Warehouse. Chronic Conditions Data Warehouse. <https://www2.ccwdata.org/web/guest/condition-categories-chronic>. Other chronic health, mental health, and potentially disabling conditions - Chronic Conditions Data Warehouse. Chronic Conditions Data Warehouse. <https://www2.ccwdata.org/web/guest/condition-categories-other>

**Supplemental Table S3. Operational Definitions for Baseline Measures**

| **Variable** | **Definition** | **Data Source** |
| --- | --- | --- |
| **Demographics** | | |
| Age | Age in years on index date; continuous variable.  Year (INDEX_DT) – Year (BENE_BIRTH_DT). | Enrollment file (MBSF) |
| Sex | Sex (SEX_IDENT_CD) on index date.  1=Male, 2=Female. | Enrollment file (MBSF) |
| Race | Race on the index date (BENE_RACE_CD).  0=Unknown, 1=White, 2=Black, 3=Other, 4=Asian, 5=Hispanic, 6=North American Native. | Enrollment file (MBSF) |
| Geographic region | States (STATE_CODE) grouped into five geographic regions. 1=Midwest, 2=Northeast, 3=South, 4=West, 5=Other. | Enrollment file (MBSF) |
| Index year | Year of the index date: 2018–2021. |  |
| SVI^a^ | RPL_THEMES=Overall vulnerability; continuous variable; range from 0-1.  The 2018 CDC/ATSDR SVI data was linked to Medicare data by matching the county (FIPS) with the state/county code (STATE_CNTY_FIPS) on index month. | CDC/ATSDR SVI |
| Dual eligibility status | Dual eligibility status (DUAL_STUS_CD_XX) on index month.  1=No duals, 2=Partial duals, 3=Full duals. | Enrollment file (MBSF) |
| LIS | Low-income subsidy status (CST_SHR_GRP_CD_XX) on index month.  1=No LIS, 2=Partial LIS, 3=Full LIS. | Enrollment file (MBSF) |
| Current entitlement reason | ENTLMT_RSN_CURR.  0=Old age and survivor’s insurance (OASI)  1=Disability insurance benefits (DIB) | Enrollment file (MBSF) |
| Original entitlement reason | ENTLMT_RSN_ORIG.  0=Old age and survivor’s insurance (OASI)  1=Disability insurance benefits (DIB) | Enrollment file (MBSF) |
| **Comorbidities: identified using ICD-10 codes as defined in the CCW Chronic Conditions Algorithms^b^** | | |
| Obesity | 0=No, 1=Yes. | Medical claims |
| Diabetic nephropathy | 0=No, 1=Yes. | Medical claims |
| Diabetic neuropathy | 0=No, 1=Yes. | Medical claims |
| Diabetic retinopathy | 0=No, 1=Yes. | Medical claims |
| AMI | 0=No, 1=Yes. | Medical claims |
| AF and flutter | 0=No, 1=Yes. | Medical claims |
| HF and non-IHD | 0=No, 1=Yes. | Medical claims |
| IHD | 0=No, 1=Yes. | Medical claims |
| Stroke | 0=No, 1=Yes. | Medical claims |
| PVD | 0=No, 1=Yes. | Medical claims |
| Hyperlipidemia | 0=No, 1=Yes. | Medical claims |
| Hypertension | 0=No, 1=Yes. | Medical claims |
| Hypothyroidism | 0=No, 1=Yes. | Medical claims |
| CKD | 0=No, 1=Yes. | Medical claims |
| COPD | 0=No, 1=Yes. | Medical claims |
| Cancer (any type) | 0=No, 1=Yes. | Medical claims |
| Anxiety disorders | 0=No, 1=Yes. | Medical claims |
| PTSD | 0=No, 1=Yes. | Medical claims |
| AUD | 0=No, 1=Yes. | Medical claims |
| DUD | 0=No, 1=Yes. | Medical claims |
| OUD | 0=No, 1=Yes. | Medical claims |
| TUD | 0=No, 1=Yes. | Medical claims |
| **Medications** | | |
| Antithrombotic medications | 0=No, 1=Yes. | RX claims (PDESAF) |
| ACE inhibitors or ARBs | 0=No, 1=Yes. | RX claims (PDESAF) |
| Beta blockers | 0=No, 1=Yes. | RX claims (PDESAF) |
| Calcium channel blockers | 0=No, 1=Yes. | RX claims (PDESAF) |
| Diuretics | 0=No, 1=Yes. | RX claims (PDESAF) |
| Statins | 0=No, 1=Yes. | RX claims (PDESAF) |
| Insulin | 0=No, 1=Yes. | RX claims (PDESAF) |
| Antipsychotics | 0=No, 1=Yes. | RX claims (PDESAF) |
| Mood stabilizers | 0=No, 1=Yes. | RX claims (PDESAF) |
| Antidepressants | 0=No, 1=Yes. | RX claims (PDESAF) |
| **Physician specialties: any specialist visits occurred within 7 days prior to index date** | | |
| Cardiologist visits | AT_PHYSN_SPCLTY_CD / PRVDR_SPCLTY:  06=Cardiology  21=Cardiac Electrophysiology  31=Intensive cardiac rehabilitation  78=Cardiac surgery  C3=Interventional cardiology  C7= Advanced heart failure and transplant cardiology | Medical claims |
| Endocrinologist visits | AT_PHYSN_SPCLTY_CD / PRVDR_SPCLTY:  46=Endocrinology | Medical claims |
| Primary care visits | AT_PHYSN_SPCLTY_CD / PRVDR_SPCLTY:  01=General practice  08=Family practice  11=Internal medicine  50=Nurse practitioner | Medical claims |
| Psychiatrist visits | AT_PHYSN_SPCLTY_CD / PRVDR_SPCLTY:  26=Psychiatry  27=Geriatric Psychiatry  86=Neuropsychiatry | Medical claims |
| **HCRU** | | |
| Number of hospitalizations | Continuous variable.  Identified in the MedPar files, where one record represents one admission (SS_LS_SNF_IND_CD ≠ “N”). | Medical claims (MedPar file) |
| Number of ED visits | Continuous variable.  ED visits that resulted in hospital admission (identified in inpatient claims) were counted as hospitalizations and excluded from the ED visit count.  A unique ED visit was defined using the Yale Definition^C^. | Medical claims  (Institutional outpatient file and Carrier file) |
| Number of outpatient encounters | Continuous variable.  A unique outpatient encounter was defined as:   - Outpatient file: each unique combination of BENE_ID, CLM_THRU_DT, PRVDR_NUM, and AT_PHYSN_NPI - Carrier file: each unique combination of BENE_ID, CLM_THRU_DT, TAX_NUM, and PRF_PHYSN_NPI | Medical claims  (Institutional outpatient file and Carrier file) |
| Number of unique medications | Continuous variable.  Number of unique generic names (GNN). | RX claims (PDESAF) |

Abbreviations: ACE inhibitors or ARBs, angiotensin-converting enzyme inhibitors or angiotensin receptor blockers; AF, atrial fibrillation; AMI, acute myocardial infarction; AUD, alcohol use disorder; CKD, chronic kidney disease; COPD, chronic obstructive pulmonary disease; DUD, drug use disorder; ED, emergency department; HCRU, health care resource utilization; HF, heart failure; IHD, ischemic heart disease; Low-income subsidy, low-income subsidy; OUD, opioid use disorder; PTSD, post-traumatic stress disorder; PVD, peripheral vascular disease; SVI, Social Vulnerability Index; TUD, tobacco use disorder.

a Reference: CDC. Social Vulnerability Index. Place and Health - Geospatial Research, Analysis, and Services Program (GRASP). Published October 22, 2024. <https://www.atsdr.cdc.gov/place-health/php/svi/index.html>
b Reference: Chronic Conditions - Chronic Conditions Data Warehouse. Chronic Conditions Data Warehouse. <https://www2.ccwdata.org/web/guest/condition-categories-chronic>. Other chronic health, mental health, and potentially disabling conditions - Chronic Conditions Data Warehouse. Chronic Conditions Data Warehouse. <https://www2.ccwdata.org/web/guest/condition-categories-other>.
c Reference: Venkatesh AK, Mei H, Kocher KE, et al. Identification of Emergency Department Visits in Medicare Administrative Claims: Approaches and Implications. Acad Emerg Med. 2017;24(4):422-431. doi:10.1111/acem.13140

**Supplemental Table S4. Overview of Evidence and Regulatory Updates on the Cardiovascular Benefits of Newer Antihyperglycemic Agents, 2018–2021**

| **Date** | **Event Description** |
| --- | --- |
| 2018/01 | ADA updated its guidelines to endorse SGLT2i and GLP‑1 RA as preferred second‑line agents for patients with T2D and established atherosclerotic CVD. |
| 2018/10 | HARMONY Outcomes trial results were published, demonstrating CV benefits (reduced MACE) with albiglutide. |
| 2018/10 | The FDA approved canagliflozin to reduce the risk of heart attack, stroke, or CV death in adults with T2D and established CVD. |
| 2019/01 | DECLARE‑TIMI 58 trial results were published, suggesting a lower rate of hospitalization for HF with dapagliflozin. |
| 2019/09 | DAPA‑HF trial results were published, showing that dapagliflozin reduces the risk of worsening HF or CV death in patients with HFrEF, regardless of diabetes status. |
| 2019/11 | REWIND trial results were published, demonstrating CV benefits (reduced MACE) with dulaglutide. |
| 2020/01 | ADA updated its guidelines, reinforcing the use of SGLT2i or GLP‑1 RA in patients with T2D and established atherosclerotic CVD, high-risk indicators, established kidney disease, or HF. |
| 2020/05 | The FDA approved dapagliflozin for adults with HFrEF to reduce the risk of CV death and hospitalization for HF. |
| 2020/08 | EMPEROR‑Reduced trial results were published, showing that empagliflozin reduces the risk of CV death or HF hospitalization in patients with HFrEF, regardless of diabetes status. |
| 2021/06 | AMPLITUDE‑O trial results were published, demonstrating CV benefits (reduced MACE) with efpeglenatide. |
| 2021/08 | EMPEROR‑Preserved trial results were published, demonstrating that empagliflozin reduces the risk of CV death or HF hospitalization in patients with HFpEF. |
| 2021/08 | The FDA updated the empagliflozin label to include an indication for treating patients with HFrEF. |

Abbreviation: ADA, American Diabetes Association; CV, cardiovascular; CVD, cardiovascular disease; FDA, U.S. Food and Drug Administration; HF, heart failure; HFpEF, heart failure with preserved ejection fraction; HFrEF, heart failure with reduced ejection fraction; T2D, type 2 diabetes mellitus.

**Supplemental Table S5. Cohort Selection Attrition Table**

| **Step** | **Criteria** | **Remaining** | **Excluded** | **% from Previous Step** |
| --- | --- | --- | --- | --- |
| 1 | Individuals with at least one RX claim for SGLT2i, GLP-1 RA, or Metformin from January 1, 2018, through December 31, 2021. | 2,281,838 | – | – |
| 2 | Individuals aged ≥18 on the index date, defined as the date of the first RX claim for SGLT2i, GLP-1 RA, or Metformin. | NR* | <11 | NR* |
| 3 | Individuals had continuous health insurance enrollment (Parts A, B and D) during the 12-month baseline period prior to the index date. | 820,240 | 1,461,593 | 35.9 |
| 4 | Individuals had no evidence of metformin or typical second-line antihyperglycemic agents during the 12-month baseline period prior to the index date. | 221,306 | 598,934 | 27.0 |
| 5 | Individuals had evidence of SMI and type 2 diabetes at baseline. | 30,412 | 190,894 | 13.7 |
| 6 | Exclude: Individuals who initiated more than one antihyperglycemic medication class on the index date. | 30,098 | 314 | 99.0 |
| 7 | Exclude: Individuals had at least one diagnosis of type 1 diabetes, gestational diabetes, polycystic ovary syndrome, organ transplant, end-stage renal disease, or HIV/AIDS at baseline. | 24,772 | 5,326 | 82.3 |
| Final | Study cohort. | 24,772 | – | – |

*NR: Not reportable due to a cell count of fewer than 11.

**Supplemental Table S6. Baseline Characteristics of First-Line Antihyperglycemic Medication Initiators, Stratified by Calendar Year: SGLT2i vs Metformin**

Abbreviations: ACE inhibitors or ARBs, angiotensin-converting enzyme inhibitors or angiotensin receptor blockers; AF, atrial fibrillation; AMI, acute myocardial infarction; AUD, alcohol use disorder; CKD, chronic kidney disease; COPD, chronic obstructive pulmonary disease; CVD, cardiovascular disease; DUD, drug use disorder; ED, emergency department; HCRU, health care resource utilization; HF, heart failure; IHD, ischemic heart disease; LIS, low-income subsidy; OUD, opioid use disorder; PTSD, post-traumatic stress disorder; PVD, peripheral vascular disease; SD, standard deviation; SMI, severe mental illness; SVI, Social Vulnerability Index; TUD, tobacco use disorder.

Geographic region: The “Other” category had fewer than 11 observations and is therefore not reportable.

**Supplemental Table S7. Baseline Characteristics of First-Line Antihyperglycemic Medication Initiators, Stratified by Calendar Year: GLP-1 RA vs Metformin**

Abbreviations: ACE inhibitors or ARBs, angiotensin-converting enzyme inhibitors or angiotensin receptor blockers; AF, atrial fibrillation; AMI, acute myocardial infarction; AUD, alcohol use disorder; CKD, chronic kidney disease; COPD, chronic obstructive pulmonary disease; CVD, cardiovascular disease; DUD, drug use disorder; ED, emergency department; HCRU, health care resource utilization; HF, heart failure; IHD, ischemic heart disease; LIS, low-income subsidy; OUD, opioid use disorder; PTSD, post-traumatic stress disorder; PVD, peripheral vascular disease; SD, standard deviation; SMI, severe mental illness; SVI, Social Vulnerability Index; TUD, tobacco use disorder.

Geographic region: The “Other” category had fewer than 11 observations and is therefore not reportable.

**Supplemental Table S8. Propensity Score Summary Statistics, 2018–2021**

| **SGLT2i (Exposed=1) versus Metformin (Exposed=0)** | | | | | | | | |
| --- | --- | --- | --- | --- | --- | --- | --- | --- |
| Year | 2018 | 2018 | 2019 | 2019 | 2020 | 2020 | 2021 | 2021 |
| Exposed | 0 | 1 | 0 | 1 | 0 | 1 | 0 | 1 |
| N Obs | 6870 | 220 | 5962 | 295 | 4406 | 305 | 3884 | 500 |
| Mean | 0.0287 | 0.1036 | 0.0430 | 0.1310 | 0.0576 | 0.1673 | 0.0947 | 0.2640 |
| Std Dev | 0.0430 | 0.0942 | 0.0558 | 0.1148 | 0.0665 | 0.1526 | 0.1071 | 0.1913 |
| Minimum | 0.0003 | 0.0022 | 0.0008 | 0.0067 | 0.0012 | 0.0055 | 0.0020 | 0.0069 |
| Maximum | 0.4348 | 0.4663 | 0.6104 | 0.6135 | 0.5345 | 0.6855 | 0.8288 | 0.8331 |
| 1st Pctl | 0.0011 | 0.0040 | 0.0027 | 0.0077 | 0.0049 | 0.0128 | 0.0052 | 0.0099 |
| 5th Pctl | 0.0024 | 0.0075 | 0.0052 | 0.0147 | 0.0090 | 0.0186 | 0.0108 | 0.0304 |
| 25th Pctl | 0.0070 | 0.0250 | 0.0131 | 0.0359 | 0.0200 | 0.0470 | 0.0288 | 0.1095 |
| Median | 0.0133 | 0.0750 | 0.0238 | 0.0999 | 0.0352 | 0.1032 | 0.0559 | 0.2205 |
| 75th Pctl | 0.0288 | 0.1564 | 0.0459 | 0.1928 | 0.0671 | 0.2514 | 0.1167 | 0.3949 |
| 95th Pctl | 0.1164 | 0.2765 | 0.1580 | 0.3742 | 0.1879 | 0.4755 | 0.3180 | 0.6386 |
| 99th Pctl | 0.2171 | 0.4156 | 0.2848 | 0.4578 | 0.3590 | 0.6375 | 0.5197 | 0.7811 |
| **GLP-1 RA (Exposed=1) versus Metformin (Exposed=0)** | | | | | | | | |
| Year | 2018 | 2018 | 2019 | 2019 | 2020 | 2020 | 2021 | 2021 |
| Exposed | 0 | 1 | 0 | 1 | 0 | 1 | 0 | 1 |
| N Obs | 6870 | 624 | 5962 | 627 | 4406 | 490 | 3884 | 565 |
| Mean | 0.0685 | 0.2453 | 0.077 | 0.2678 | 0.0773 | 0.3045 | 0.1014 | 0.3028 |
| Std Dev | 0.099 | 0.1773 | 0.1079 | 0.1876 | 0.1121 | 0.2192 | 0.1173 | 0.2231 |
| Minimum | 0.0008 | 0.0065 | 0.0015 | 0.0098 | 0.0004 | 0.0068 | 0.0021 | 0.019 |
| Maximum | 0.7645 | 0.8327 | 0.7892 | 0.7765 | 0.8461 | 0.9136 | 0.8049 | 0.8546 |
| 1st Pctl | 0.0028 | 0.0118 | 0.0044 | 0.016 | 0.0031 | 0.0143 | 0.0049 | 0.0255 |
| 5th Pctl | 0.0059 | 0.0242 | 0.0078 | 0.0298 | 0.0063 | 0.0291 | 0.0103 | 0.0412 |
| 25th Pctl | 0.016 | 0.088 | 0.0188 | 0.0982 | 0.0176 | 0.1012 | 0.0309 | 0.1134 |
| Median | 0.0304 | 0.2221 | 0.0356 | 0.2528 | 0.0363 | 0.2781 | 0.0612 | 0.2386 |
| 75th Pctl | 0.0679 | 0.3584 | 0.075 | 0.3982 | 0.0762 | 0.4672 | 0.1223 | 0.4616 |
| 95th Pctl | 0.29 | 0.5784 | 0.3298 | 0.5977 | 0.3207 | 0.6986 | 0.3525 | 0.7285 |
| 99th Pctl | 0.4915 | 0.7043 | 0.5261 | 0.7105 | 0.5801 | 0.8459 | 0.5872 | 0.8244 |

**Supplemental Figure S1. Trends in Standardized Mean Differences for Selected Baseline Characteristics, 2018–2021**

Abbreviations: CKD, chronic kidney disease; CVD, cardiovascular disease; HF, heart failure; SMD, standardized mean differences.

Figure legend:

- SMDs were calculated pairwise, comparing SGLT2i vs metformin and GLP-1 RA vs metformin, with metformin as the reference group.
- Positive SMDs indicate higher prevalence (or mean values) among SGLT2i (or GLP-1 RA) initiators; negative SMDs indicate higher prevalence (or mean values) among metformin initiators.
- By convention, an absolute SMD <0.1 was considered to indicate a negligible difference between groups.
- We defined comorbidities based on diagnoses recorded during the 365-day baseline period; we defined specialty visits as any visit to the specified specialist within 7 days before the index date.
